# Sterol Carrier Protein-2, a Nonspecific Lipid-Transfer Protein, in Intracellular Cholesterol Trafficking in Testicular Leydig Cells

**DOI:** 10.1371/journal.pone.0149728

**Published:** 2016-02-22

**Authors:** Nancy C. Li, Jinjiang Fan, Vassilios Papadopoulos

**Affiliations:** 1 The Research Institute of the McGill University Health Centre, Center for Translational Biology, 1001 Boul. Décarie, Montreal, Quebec, H4A 3J1, Canada; 2 Department of Pharmacology & Therapeutics, McGill University, Montreal, Quebec, H3G 1A4, Canada; 3 Department of Medicine, McGill University, Montreal, Quebec, H3G 1A4, Canada; 4 Department of Biochemistry, McGill University, Montreal, Quebec, H3G 1A4, Canada; University of Hull, UNITED KINGDOM

## Abstract

Sterol carrier protein-2 (SCP2), also called nonspecific lipid-transfer protein, is thought to play a major role in intracellular lipid transport and metabolism, and it has been associated with diseases involving abnormalities in lipid trafficking, such as Zellweger syndrome. The *Scp2* gene encodes the 58 kDa sterol carrier protein-x (SCPX) and 15 kDa pro-SCP2 proteins, both of which contain a 13 kDa SCP2 domain in their C-termini. We found that 22-NBD-cholesterol, a fluorescent analog of cholesterol and a preferred SCP2 ligands, was not localized in the peroxisomes. This raises questions about previous reports on the localization of the SCPX and SCP2 proteins and their relationship to peroxisomes and mitochondria in intracellular cholesterol transport. Immunofluorescent staining of cryosections of mouse testis and of MA-10 mouse tumor Leydig cells showed that SCPX and SCP2 are present in both mouse testicular interstitial tissue and in MA-10 cells. Fluorescent fusion proteins of SCPX and SCP2, as well as confocal live-cell imaging, were used to investigate the subcellular targeting of these proteins and the function of the putative mitochondrial targeting sequence. The results showed that SCPX and SCP2 are targeted to the peroxisomes by the C-terminal PTS1 domain, but the putative N-terminal mitochondrial targeting sequence alone is not potent enough to localize SCPX and SCP2 to the mitochondria. Homology modeling and molecular docking studies indicated that the SCP2 domain binds cholesterol, but lacks specificity of the binding and/or transport. These findings further our understanding of the role of SCPX and SCP2 in intracellular cholesterol transport, and present a new point of view on the role of these proteins in cholesterol trafficking.

## Introduction

Sterol carrier protein-2 (SCP2), also known as nonspecific lipid transfer protein, is a lipid-binding protein associated with many proposed functions in lipid metabolism. The *Scp2* gene contains two transcription initiation sites, giving rise to the 58 kDa sterol carrier protein-x (SCPX) and 15 kDa pro-SCP2 proteins, both of which contain the complete 13 kDa SCP2 protein or domain sequence in their C-termini [1,2]. SCPX is partially cleaved to form a 46 kDa 3-ketoacyl CoA thiolase and the 13 kDa SCP2 [3], while the 15 kDa pro-SCP2 is post-translationally processed to yield the mature 13 kDa SCP2 [4]. SCP2 is likely involved in lipid trafficking and metabolism in general, as it has been found to bind to and participate in the metabolism of fatty acids [5–7], fatty acyl-CoAs [8,9], cholesteryl esters [10], phospholipids [11–13], and various other lipids. SCP2 is thought to be a soluble sterol carrier because of its ability to bind to sterols and its localization to multiple organelles, such as the peroxisomes, mitochondria, endoplasmic reticulum, and cytosol [14–16].

SCP2, as a lipid transfer protein, has been shown to play a key role in the intracellular movement of cholesterol [17–19] by transporting cholesterol from intracellular sites, such as lipid droplets, to the mitochondria, where the first committed step of steroidogenesis occurs [20]. In the Leydig cells, the actions of SCP2 in the peroxisomes are thought to be critical in testosterone production [21]. Changes in SCP2 levels or lack of SCP2 expression have been associated with abnormalities in the intracellular trafficking and metabolism of cholesterol and other lipids [22,23]. Thus, further investigation into the intracellular actions of SCP2 might lead to a better understanding of the pathology and possible pharmacological avenues that can be used to treat these lipid-associated abnormalities.

The motivation for this research comes from our preliminary observations that 22-NBD-cholesterol did not localize to the peroxisomes of MA-10 cells (see below). This finding is unexpected, as previous studies have established that SCP2 is able to bind 22-NBD-cholesterol with high affinity [24], and that SCP2 is primarily targeted to the peroxisomes [14–16]. Taken together, these two pieces of evidence raised questions concerning whether SCP2 is involved in the intracellular targeting of 22-NBD-cholesterol. Given the novelty of these preliminary observations and the evidence that SCPX and SCP2 play an important role in steroid biosynthesis, we were encouraged to investigate the possible reasons behind the lack of peroxisomal localization of 22-NBD-cholesterol to better understand the functions of these proteins.

The actions of SCPX and SCP2 in the peroxisomes and mitochondria are of particular interest in our investigation. The C-termini of the SCPX and SCP2 proteins contain a peroxisomal targeting PTS1 signal [25], while the N-termini demonstrate characteristics typical of a predicted mitochondrial targeting sequence [26]. It has been observed that the 20 amino acid N-terminal sequence found in the 15 kDa pro-SCP2 contains features similar to those of known mitochondrial targeting sequences [17]. However, the functionality of this putative mitochondrial targeting sequence remains to be experimentally validated.

The goal of this project is to further investigate the subcellular targeting of SCPX and SCP2, specifically the likely dual targeting of these proteins to peroxisomes and mitochondria, and the role that this targeting plays in the intracellular trafficking of cholesterol. The subcellular localization of SCPX and SCP2 was investigated by creating fluorescent fusion proteins tagged on either the C-terminal or N-terminal end, which contain putative peroxisomal and mitochondrial targeting sequences, respectively. In addition, we examined the distribution of SCPX and SCP2 proteins in MA-10 cells and mouse testes, the function of the putative mitochondrial targeting sequence, and the characteristics of cholesterol binding to SCP2. We hypothesize that both SCPX and SCP2 will be targeted to the peroxisomes and/or mitochondria, and that SCPX and SCP2 will bind cholesterol and assist in nonvesicular sterol transport and intracellular distribution.

## Materials and Methods

### Materials

The original, full-length cDNA clone of the *Scp2* gene was purchased as the EST clone from GE Healthcare Dharmacon Inc. (Lafayette, CO, USA), and the subcloning of the gene into the appropriate vectors was performed in the laboratory. The antibodies used in this study were the SCP2 rabbit polyclonal antibody specific to the 15 kDa SCP2 protein (Proteintech^™^: 23006-1-AP; Chicago, IL, USA); rabbit monoclonal anti-sterol carrier protein 2 antibody specific to the 58 kDa SCPX protein (EPR9022; Abcam: ab140126; Toronto, ON, Canada); rabbit polyclonal anti-COX IV antibody (mitochondrial loading control; Abcam: ab16056; Toronto, ON, Canada); rabbit polyclonal anti-VDAC1/porin antibody (mitochondrial loading control; Abcam: ab15895; Toronto, ON, Canada); rabbit polyclonal anti-HSP60 antibody (H-300) (mitochondrial loading control; Santa Cruz Biotechnology: sc-13966; Dallas, TX, USA); rabbit polyclonal anti-CAT (catalase) antibody (H-300) (peroxisomal marker; Santa Cruz Biotechnology: sc-50508; Dallas, TX, USA); rabbit polyclonal anti-glyceraldehyde 3-phosphate dehydrogenase (GAPDH) antibody (protein loading control; Trevigen: 2275-PC-100; Gaithersburg, MD, USA); mouse monoclonal anti-PMP70 antibody (Sigma-Aldrich: SAB4200181; Oakville, ON, Canada); mouse anti-VDAC1/porin antibody (20B12AF2; Abcam: ab14734; Toronto, ON, Canada); Alexa Fluor^®^ 546 donkey anti-rabbit immunoglobulin G (IgG) (Molecular Probes, Burlington, ON. Canada); anti-rabbit IgG horseradish peroxidase-linked antibody (Cell Signalling Technology, Danvers, MA, USA); and Pacific Blue^™^ goat anti-mouse IgG (H+L), highly cross-adsorbed (Life Technologies: P31582; Burlington, ON. Canada). Mito-DsRed-pero, a dual subcellular targeting protein marker that localizes to both the mitochondria and peroxisomes, was made locally by fusing the ACBD2–mitochondrial targeting presequence to the N-terminus of the DsRed fluorescent protein (DsRed) and the peroxisomal targeting PTS1 signal peptide to the C-terminus of the DsRed; DsRed-pero, DsRed with a C-terminal PTS1 signal peptide; and TSPO-DsRed, mouse translocator protein fused at its C-terminus to DsRed (Fan et al, unpublished). 22-NBD-cholesterol (22-(N-(7-Nitrobenz-2-Oxa-1,3-Diazol-4-yl)Amino)-23,24-Bisnor-5-Cholen-3β-Ol) was purchased from Life Technologies (Burlington, ON. Canada).

### Sequence selection, alignment, and RNA sequencing (RNA-seq)

The mouse SCPX sequence was originally obtained from the EST clone (MMM1013-202763426, Dharmacon, Pittsburgh, PA, USA), and it was then subcloned to make the SCPX and SCP2 fusion proteins. The orthologous human *SCPX* gene was aligned with the mouse *Scpx* using Cluster X, and variable sequence features were retrieved from the literature. As for the RNA-seq, total RNA from MA-10 cells was extracted using Trizol Reagent (Invitrogen; Thermo Fisher Scientific, Waltham, MA, USA), an Illumina HiSeq 2000 paired-end (2×100 bp) library was prepared, and then the total transcripts of the cells was sequenced at the McGill University and Genome Quebec Innovation Center [27]. Data analysis was performed using the RNA-Seq analysis pipeline in the Genome Analysis ToolKit from the Center. The RNA-Seq data was mapped and presented using the SeqMonk software package (http://www.bioinformatics.bbsrc.ac.uk/projects/seqmonk/; Babraham Bioinformatics, Cambridge, UK) and the UCSC Genome Browser (http://genome.ucsc.edu) with a custom track.

### Immunofluorescent staining with frozen mouse tissue sections and cells

For the immunofluorescent staining of mouse tissue sections, wild-type mouse testes were embedded in optimum cutting temperature (OCT) medium, and 6 μm cryosections were prepared at the Histology Core Facility of the Goodman Cancer Research Centre (McGill University, Montreal, QC, Canada). The staining was performed using a method similar to that described previously [28]. The cryosections were incubated with antibodies specific for either SCPX or SCP2, followed by fluorescence-conjugated Alexa Fluor^®^ 546 donkey anti-rabbit IgG (Molecular Probes, Burlington, ON. Canada). Images were obtained using an Olympus Fluoview FV1000 confocal laser scanning microscope and an inverted fluorescence microscope (Olympus IX5; Olympus Corporation, Tokyo, Japan) under the same conditions. The mice used in this study were C57BL/6J purchased from the Jackson Laboratory (https://www.jax.org), and housed in the animal facilities at the McGill University Health Centre (RI-MUHC, Montreal, Quebec), and all of the husbandry procedures were performed following the standard protocols. Two-month-old wild type mice were sacrificed by CO_2_ asphyxiation and the relevant tissues were collected for cryosectioning. All animal studies and procedures were approved by the McGill University Animal Care and Use Committee, and performed in accordance with the recommendations in the Canadian Council of Animal Care Guidelines.

In preparation for the immunofluorescent staining of cells, MA-10 cells were grown on a 35 mm FluoroDish^™^ sterile culture dish (World Precision Instruments, Sarasota, FL, USA). Cells were washed with 1X phosphate buffered saline (PBS), fixed using 4% PFA solution for 20 minutes, permeabilized using 0.1% Triton X-100 in PBS for 1 minute, and incubated in a blocking solution composed of 1% bovine serum albumin (BSA) in PBS for 1 hour. Cells were incubated with rabbit antibodies specific to SCPX or SCP2, and PMP70 or VDAC1 overnight at 4°C. The following day, the cells were washed with 1X PBS and incubated with fluorescence-conjugated Alexa Fluor 546 donkey anti-rabbit IgG (Molecular Probes; Burlington, ON. Canada) and Pacific Blue^™^ goat anti-mouse IgG for 1 hour at room temperature. The nuclei were counterstained using UltraCruz^™^ mounting medium (Santa Cruz Biotechnology: sc-24941; Dallas, TX, USA). Cells were observed under an Olympus Fluoview FV1000 confocal laser scanning microscope with the following excitation/emission wavelengths: red (DsRed), 557/592 nm; and blue (DAPI), 350/460 nm. For a better view of the colocalization, the images in the blue channel were painted green.

### Subcellular fractionation, protein extraction, SDS-PAGE gel electrophoresis and immunoblotting techniques

For the subcellular fractionation, MA-10 cells were grown in 150-mm petri dishes to 70% confluence, washed with PBS, and harvested for further processing [29]. In brief, to prepare crude membranes, cells were homogenized with a glass potter followed by 3 cycles of freezing and thawing in isolation buffer 1 (225 mM mannitol, 75 mM sucrose and 30 mM Tris.HCI, pH 7.4). Homogenates were centrifuged at 800 x g for 5 minutes at 4°C. The collected supernatants (containing cytosolic proteins) were centrifuged twice at 11 000 x g for 10 minutes at 4°C to obtain crude mitochondria. These preparations were resuspended in isolation buffer 2 (250mM mannitol, 5mM HEPES [pH 7.4] and 0.5mM EGTA). To confirm the enrichment of the crude mitochondrial fraction, we performed immunoblot analysis using anti-COX IV, anti-VDAC1 and anti-HSP60 antibodies as mitochondrial loading controls, anti-CAT (catalase) antibody as a peroxisomal marker, and anti-GAPDH antibody as a whole protein loading control.

For protein extraction, MA-10 cells were grown in six-well Cell Culture Cluster dishes (Corning Incorporated, Corning, NY, USA), and the total protein of the cells was harvested using 250 μL of Mammalian Protein Extraction Reagent (M-PER) (Thermo Fisher Scientific, Burlington, ON, Canada) per well, followed by centrifugation at 14,000 x g for 10 minutes at 4°C. The protein concentration was measured using the Bradford dye assay (Bio-Rad Laboratories Inc., Hercules, CA, USA) following the manufacturer's instructions, and absorbance was measured at 595 nm. Sodium dodecyl sulfate (SDS)-polyacrylamide gel electrophoresis (PAGE) and immunoblot analysis were performed using a procedure similar to that previously described [27]. Briefly, 25 μg of total protein extract was electrophoretically separated on a 4%–20% Tris–glycine gradient gel, transferred to a polyvinylidene fluoride (PVDF) membrane, and blocked overnight in 10% skim milk dissolved in 1X TBST buffer (50 mM Tris, 150 mM NaCl, 0.05% Tween 20) at 4°C. The membranes were cut horizontally to visualize GAPDH levels as a loading control with the locations of the cuts guided by the Novex Sharp Pre-Stained Protein Standard (Thermo Fisher Scientific, Burlington, ON, Canada). Membranes were incubated with primary antibodies specific to SCPX, SCP2, and GAPDH, followed by the secondary antibody, anti-rabbit IgG horseradish peroxidase-linked antibody (Cell Signaling Technology, Danvers, MA, USA). The proteins were then visualized using the Amersham ECL Western Blotting Detection Reagent (GE Healthcare Life Sciences, Mississauga, ON, Canada) and images were captured using a FUJI image reader LAS4000 (Fujifilm, Tokyo, Japan).

### Plasmid construction

The ECFP-Bak (Plasmid 31501; the ECFP-N1 with Bak protein) vector (Addgene, Cambridge, MA, USA) was used to create the plasmids that expressed N-terminal tagged SCPX and SCP2 (pECFP-SCPX and pECFP-SCP2), while the pECFP-C1 vector (Clontech Laboratories, Mountain View, CA, USA) was used to create the C-terminal tagged SCPX and SCP2 (pSCPX-ECFP and pSCP2-ECFP). The pEGFP-N1 vector (Clontech Laboratories, Mountain View, CA) was used to create fluorescent fusion proteins of the putative mitochondrial targeting sequences of SCPX and SCP2 (pSCPX-mito-EGFP and pSCP2-mito-EGFP). Table 1 lists the forward and reverse primers (Integrated DNA Technologies, Coralville, IA, USA), restriction enzymes (New England Biolabs, Ipswich, MA, USA), and antibiotics (Sigma-Aldrich Co., St Louis, MO, USA) used during the construction of each plasmid. Polymerase chain reaction (PCR) was performed to amplify the SCPX and SCP2 sequences and to create the appropriate restriction enzyme sites surrounding the desired sequence. The appropriate restriction enzymes (Table 1) were used to digest both the vectors and inserts, and ligation was performed using the Rapid DNA Ligation Kit (Hoffman-La Roche Ltd., Basel, Switzerland). Ligated vectors were transformed into Subcloning Efficiency^™^ DH5α^™^ Competent *Escherichia coli* Cells (Life Technologies; Thermo Fisher Scientific, Burlington, ON, Canada), and *E*. *coli* cells that had been successfully transformed were selected for using the appropriate antibiotic (Table 1). PCR was performed to verify the presence of the desired DNA sequence in the vectors. Plasmids were isolated using the QIAprep Spin Miniprep Kit (Qiagen, Venlo, The Netherlands) and sequenced using Sanger DNA sequencing (Genome Quebec Innovation Centre, McGill University, Montreal, QC, Canada) to confirm that no mutations were introduced. Plasmids were then amplified by growing transformed *E*. *coli* cells overnight in Luria Broth (LB) followed by isolating nucleic acids using the HiSpeed Plasmid Maxi Kit or the QIAprep Spin Miniprep Kit (Qiagen, Venlo, The Netherlands).

**Table 1:** The primer sequences, restriction enzymes, and antibiotics used in the construction of plasmids containing the *Scpx* and *Scp2* gene sequences.

Vector	Primer Sequence	Restriction Enzyme	Antibiotic
ECFP-Bak(eCFP-SCPX/2)	Reverse Primer (SCPX): GCGCTCGAGTGCCTTCTGTCGCTTTGAAATC	XhoI	Kanamycin
	Reverse Primer (SCP2): GCGCTCGAGTGGGTTTTCCCGAAGCTGC	XhoI	
	Forward Primer (SCPX/2): CGCGAATTCTCACAGCTTAGCTTTGCCCGG	EcoRI	
pECFP-C1(SCPX/2-eCFP)	Reverse Primer (SCPX): GCGCTCGAGATGCCTTCTGTCGCTTTGAAATC	XhoI	Kanamycin
	Reverse Primer (SCP2): GCGCTCGAGATGGGTTTTCCCGAAGCTGC	XhoI	
	Forward Primer (SCPX/2): CGCGAATTCGCAGCTTAGCTTTGCCCGG	EcoRI	
	SCP2-seq-F Primer^a^: AGCTGGCAGCTTCGGGAAAACC		
pEGFP-N1(SCPX/2-mito-EGFP)	Mito-seqx-HindIII: GCTCAAGCTTATGCCTTCTGTCGCTTTG	HindIII	Kanamycin
	Mitoseqx-KpnI CGCGGTACCGCGGTCATGCCAACGCCGACC	KpnI	
	Mito-seq2-HindIII GCTCAAGCTTATGGGTTTTCCCGAAGCTGC	HindIII	
	Mitoseq2-KpnI CGCGGTACCGCGGTGGGAGCAGCTGAAACC	KpnI	

^a^Note: SCP2-seq-F Primer was used for Sanger DNA sequencing of the middle portion of the SCPX clone.

### Cell culture and transfection

MA-10 mouse Leydig tumor cells (kindly provided by Dr M Ascoli, University of Iowa, Ames, IA, USA) were grown in Dulbecco’s Modified Eagle’s Medium (DMEM)/Ham F12 (50:50) (Gibco^®^; Thermo Fisher Scientific, Burlington, ON, Canada), supplemented with 5% fetal bovine serum (FBS), and 2.5% horse serum (HS). The MA-10 cells were incubated at 37°C and 3.7% CO_2_. Cells were transfected with 4 μg of plasmid according to the method previously described using the Opti-MEM Reduced Serum Medium (Life Technologies; Thermo Fisher Scientific, Burlington, ON, Canada) and Lipofectamine 2000 Transfection Reagent (Thermo Fisher Scientific, Burlington, ON, Canada) [30].

### Live-cell laser confocal microscopy

MA-10 cells grown on a 35 mm FluoroDish^™^ sterile culture dish (World Precision Instruments, Sarasota, FL, USA) were co-transfected with the mito-DsRed-pero plasmid and one of the N-terminal or C-terminal fusion proteins of SCPX or SCP2. In addition, to study the potential roles of the predicted mitochondrial targeting sequences (presequences) of SCPX and SCP2, the two presequences with lengths of 66 bp and 60 bp, respectively, were cloned into pEGFP-N1 vector to produce two plasmids: pSCPX-mito-EGFP and pSCP2-mito-EGFP. Each of these plasmids was co-transfected into MA-10 cells with ACBD2-mito-DsRed as the mitochondrial marker. Images were obtained using an Olympus Fluoview FV1000 laser confocal microscope (UPLSAP, ×100) to observe the presence and subcellular location of the proteins.

### Protein homology modeling and protein small-molecule docking

Putative 3D structures of the mouse SCP2 domain were predicted via an automated comparative protein modeling server (Swiss-model; http://www.expasy.ch) with the optimized mode using the coordinates of three structures, respectively: an X-ray crystallography structure of the rabbit SCP2 (PBD: 1C44), a nuclear magnetic resonance (NMR) structure of the human SCP2 (PBD: 1QND), and an X-ray crystallography structure of the ligand-bound SCP2-like domain of human peroxisomal multifunctional enzyme type 2 (MFE-2; PDB: 1IKT). A protein small-molecule docking analysis was performed using AutoDock Vina [31], and the ligand PDB coordinates for cholesterol and 22-NBD-cholesterol were obtained from ChemSpider (http://www.chemspider.com) and the ChemicalBook (http://www.chemicalbook.com) respectively. The modeling was viewed using the Swiss PDB Viewer (V. 4.10; http://spdbv.vital-it.ch).

## Results

### 22-NBD-cholesterol does not localize to peroxisomes

**Fig 1:**
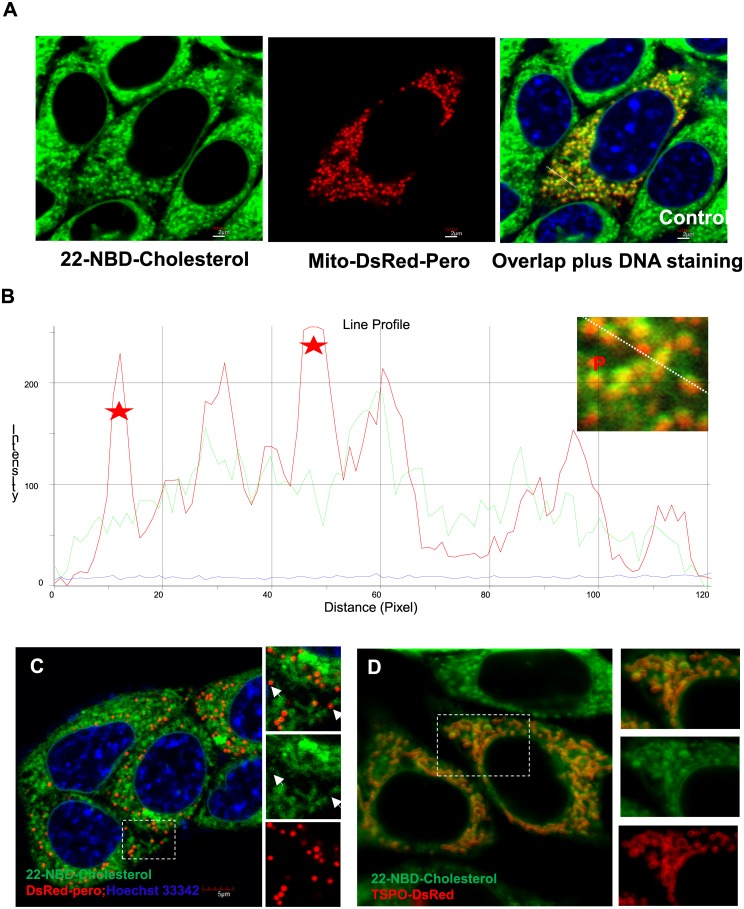
Subcellular localization of 22-NBD-cholesterol with mitochondria and peroxisomes. (A) Confocal laser microscopy images of MA-10 cells stained with 22-NBD-cholesterol (left), transfected with mito-DsRed-pero (center), and the overlap of 22-NBD-cholesterol and mito-DsRed-pero staining (right). (B) Comparison of the fluorescence intensity profiles of the mitochondria and peroxisomes (P) in red, and of 22-NBD-cholesterol in green. 22-NBD-cholesterol (green) does not localize with the mito-DsRed-pero tagged peroxisomes, shown as the small high-intensity dots (red), in the MA-10 cell line. The areas where the fluorescence intensities of the 22-NBD-cholesterol and peroxisomes do not correlate are marked by red stars. (C) Colocalization of 22-NBD-cholesterol (green) with DsRed-pero (red), a peroxisomal marker. The panels on the right of the image show the highlighted region with overlapped green and red channels, the green channel only, and the red channel only (from top to bottom). (D) Colocalization of 22-NBD-cholesterol (green) with TSPO-DsRed (red), a mitochondrial marker. The panels on the right of the image show the highlighted region with overlapped green and red channels, the green channel only, and the red channel only (from top to bottom).

22-NBD-cholesterol is commonly used as the fluorescent cholesterol analog for fluorescence imaging of natural cholesterol, and it has been shown to bind to SCP2 with high affinity [24]. Given that SCP2 is primarily targeted to the peroxisomes [14–16], it is suspected that SCP2 would mediate the intracellular targeting of 22-NBD-cholesterol to the peroxisomes. It has previously been demonstrated that the peroxisomes are important for steroid biosynthesis, as they contain a number of enzymes involved in the initial steps of cholesterol synthesis. Further supporting this point is the observation that patients with peroxisomal deficiency diseases have impaired cholesterol synthetic ability [32,33]. MA-10 cells were treated with 22-NBD-cholesterol, peroxisomes and mitochondria were labeled using the mito-DsRed-pero tag, and live-cell imaging was performed to investigate the subcellular localization of 22-NBD-cholesterol (Fig 1A). Fluorescence intensity was measured using the ImagePro Plus software (Media Cybernetics, Rockville, MD, USA), with the region of interest (ROI) drawn through a peroxisome, detected using the mito-DsRed-pero tag (Fig 1B). It was surprisingly observed that the fluorescence of the 22-NBD-cholesterol did not colocalize with the peroxisomes, which indicates that 22-NBD-cholesterol does not localize to the peroxisomes in MA-10 cells. To validate our observation, MA-10 cells were co-transfected with either DsRed-pero—a peroxisomal marker (Fig 1C), or TSPO-DsRed—a mitochondrial marker (Fig 1D), separately. It is clear that 22-NBD-cholesterol is only localized at mitochondria, as shown by its co-localization with TSPO-DsRed; the translocator protein (TSPO) is an outer mitochondrial membrane protein [34]. Several questions raised herein from these observations will be addressed in detail in this study.

### Putative amino acid sequence alignment and predicted mitochondrial targeting sequences of SCPX and SCP2

**Fig 2:**
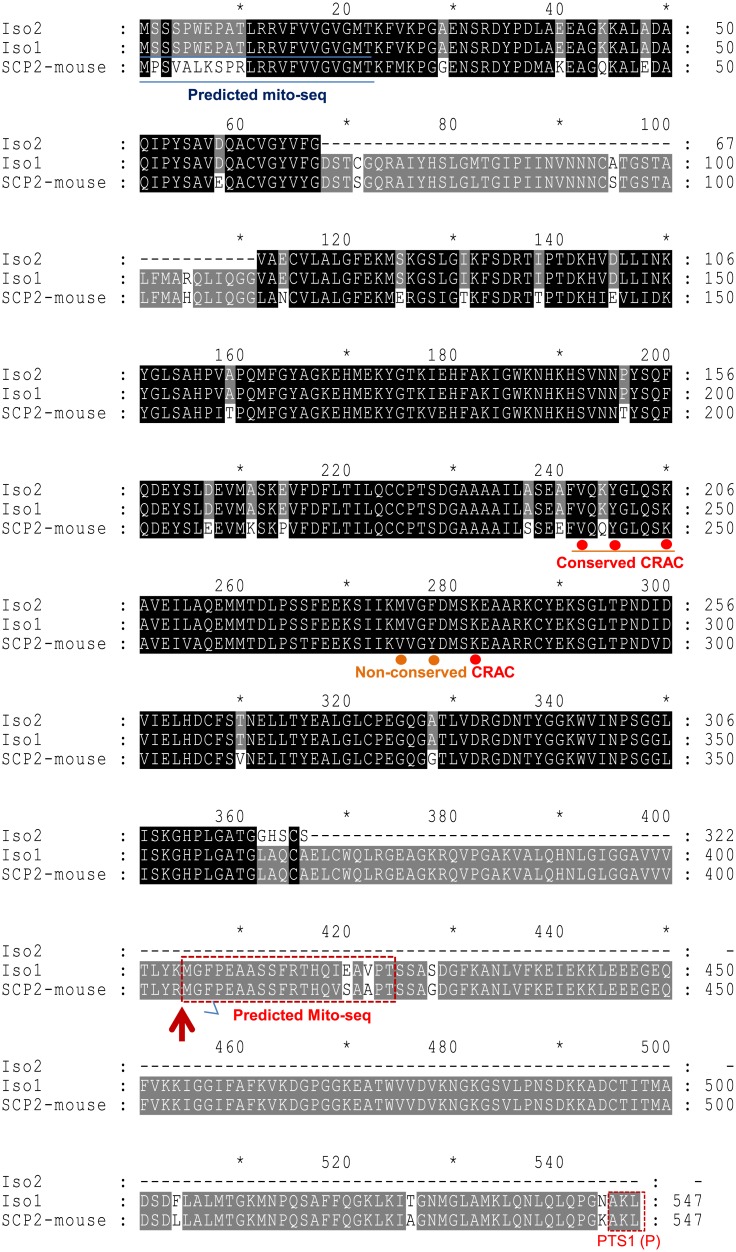
Sequence alignment and analysis of the predicted amino acid sequences of the mouse *Scp2* gene and isoforms 1 and 2 of the human *Scp2* gene. The sequence alignment shows that the *Scp2* gene encodes the SCPX and pro-SCP2 proteins, both of which contain the sequence for the SCP2 protein or domain at the C-terminal end. The predicted mitochondrial targeting sequences (mito-seqs or presequences), peroxisomal targeting PTS1 signal (PTS1 [P]), and CRAC motifs following a L/V-(X)1–5-Y-(X)1–5-R/K pattern in SCPX and SCP2 are indicated. The red upward arrow indicates the start of the predicted SCP2 sequence (GenBank ID: M62361 for mouse and M55421 for human), containing a proposed mito-seq highlighted in the dotted square.

The nucleic acid sequences and their corresponding predicted amino acid sequences were retrieved from the GenBank (accession numbers: M62361 and M55421) for isoforms 1 and 2 of the human *SCP2* gene, as well as for the mouse *Scp2* gene (Fig 2). The *Scp2* gene encodes the SCPX and pro-SCP2 proteins, both of which contain the sequence for the SCP2 protein or domain at the C-terminal end [25]. The B2 element possessing a pol II promoter is found just prior to the sequence encoding the SCP2 protein [27]. The C-terminal sequence contains a peroxisomal-targeting PTS1 signal [25], while the N-terminal sequence contains a putative mitochondrial targeting sequence (mito-seq), as previously reported and as predicted by the Mitoprot service (https://ihg.gsf.de/ihg/mitoprot.html) [26]. A conserved cholesterol recognition/interaction amino acid consensus (CRAC) sequence (CRAC: L/V-X [1–5]-Y-X [1–5]-R/K) and a nonconserved CRAC motif are both present in the SCPX protein, based on comparisons with the CRAC motif of the translocator protein (18-kDa), previously known as the peripheral-type benzodiazepine receptor (PBR) [35].

### Low mRNA expression of the *Scp2* gene determined by RNA-Seq

**Fig 3:**
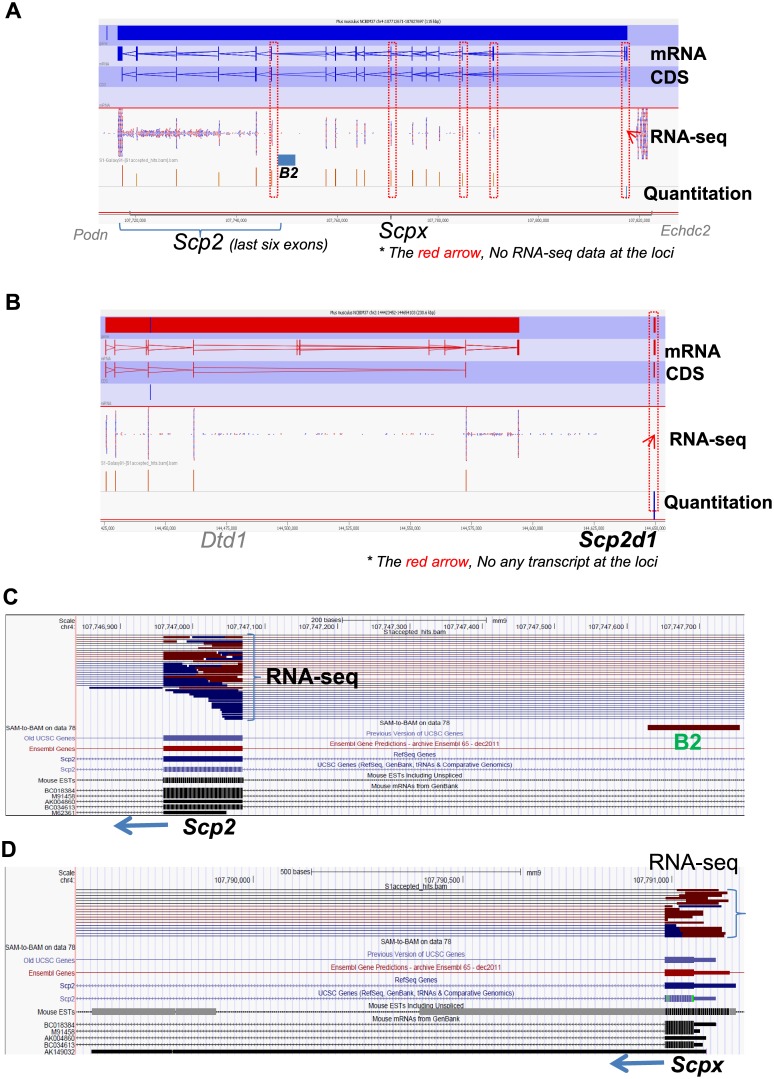
Low transcriptional expression of the *Scp2* gene in the MA-10 cell line. (A) RNA-Seq data show that the *Scp2* and *Scpx* transcripts have low expression in the MA-10 cell line. (B) No transcripts were seen at the control locus for the *Scp2d1* gene, used as a negative control in the RNA-seq. (C) A detailed look at the first exon of the *Scp2* gene shows the low expression levels of this exon, and illustrates the B2-derived promoter located just prior to the gene. (D) A detailed look at the first exon of the *Scpx* shows the low expression levels of this exon.

The entire transcriptome of the MA-10 cell line was sequenced to determine the transcription level of the *Scp2* gene. The total RNA was collected from MA-10 cells and RNA-Seq was performed using the Illumina HiSeq 2000 and MiSeq technology (Genome Quebec Innovation Centre, McGill University, QC, Canada). The RNA sequences were then mapped onto the genome using Galaxy webserver (version 15.03.1), SeqMonk (version 0.29.0), and the UCSC Genome Browser. The RNA-Seq data show that the *Scp2* and *Scpx* transcripts have relatively low expression in the MA-10 cell line (Fig 3A). As a negative control, no transcript was seen at the locus for the *Scp2d1* gene (Fig 3B). A detailed look at the first exon of the sequences giving rise to the *Scp2* (Fig 3C) and *Scpx* (Fig 3D) transcripts further demonstrates the low expression of these transcripts. The presence of the B2-derived pol II promoter, which may play a role in the transcription of *Scp2*, just prior to the *Scp2* gene, is also illustrated (Fig 3C).

### Abundant expression of SCPX and SCP2 in mouse testicular interstitial tissue

**Fig 4:**
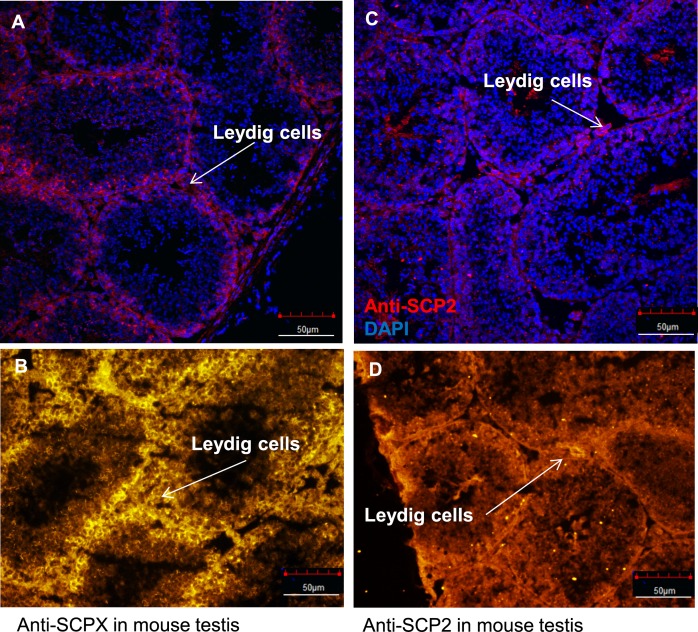
Immunofluorescent-stained cryosections of mouse testis tissues using SCPX- and SCP2-specific antibodies. The analysis was performed to investigate the endogenous distribution of SCPX and SCP2 in the wild-type mouse testis. (A–B) SCPX is highly expressed in the interstitial connective tissue of the mouse testis, an area rich in Leydig cells, but is not expressed in the germ cells in the seminiferous tubules. (C–D) SCP2 was found to have the same distribution as SCPX, but with a lower level of expression. Scale bar, 50 μm.

Immunofluorescent staining was assessed using confocal imaging and conventional epifluorescence microscopy performed to investigate the endogenous distribution of SCPX and SCP2 in a cryosection of wild-type mouse testis. It was found that SCPX is highly expressed in the interstitial connective tissue of the mouse testis, an area rich in interstitial cells of Leydig, but it is barely expressed in the germ cells of the seminiferous tubules (Fig 4A and 4B). SCP2 was found to have the same distribution as SCPX within the testis, but is expressed at a lower level (Fig 4C and 4D).

### Distribution of endogenous SCPX and SCP2 in MA-10 cells

**Fig 5:**
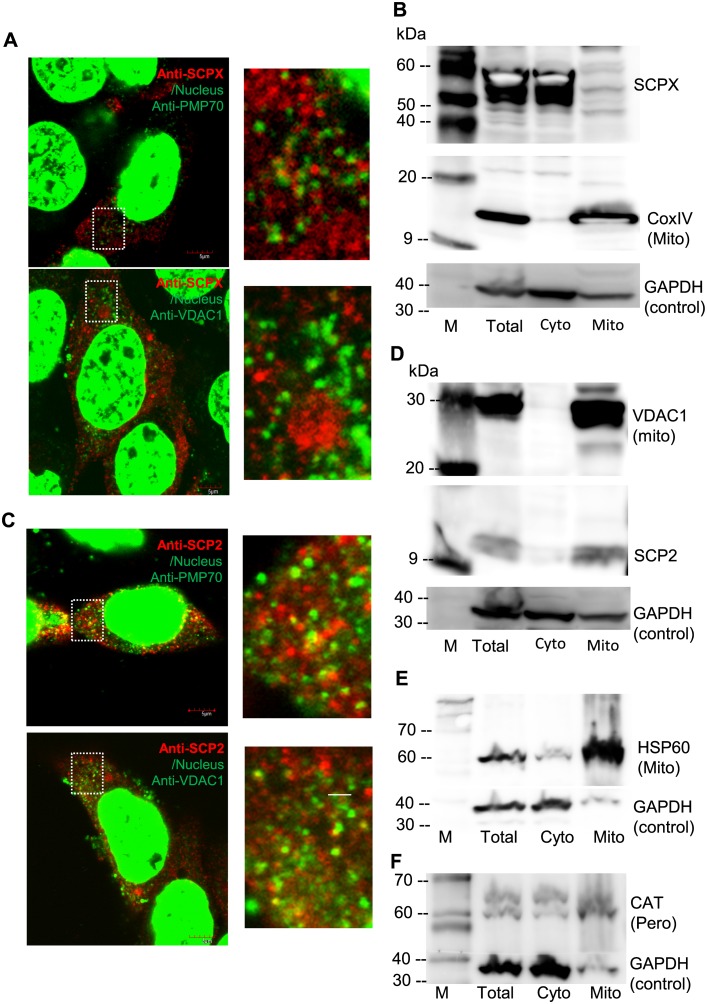
Double immunofluorescent staining and immunoblot analysis of the endogenous SCPX and SCP2 proteins in MA-10 cells. (A) Double immunofluorescent staining of endogenous SCPX in MA-10 cells (red), PMP70 (peroxisomal marker protein, painted in green), and VDAC1 (mitochondrial marker protein, painted in green). The nucleus was counterstained with DAPI (painted in green). Scale bar, 5 μm. (B) Immunoblot analysis of endogenous SCPX in subcellular fractions of MA-10 cell lysates. COXIV is used as a mitochondrial marker protein, and GAPDH is used as a whole cell lysate loading control. M, the protein marker, indicated in kDa; Total, whole cell lysate; Cyto, cytosolic fraction; Mito, mitochondrial-enriched fraction. (C) Double immunofluorescent staining of endogenous SCP2 in MA-10 cells (red), PMP70 (painted in green), and VDAC1 (painted in green). The nNucleus was counterstained with DAPI (painted in green). Scale bar, 5 μm. (D) Immunoblot analysis of endogenous SCP2 in subcellular fractions of MA-10 cell lysates. VDAC1 is used as a mitochondrial marker protein, and GAPDH is used as a whole cell lysate loading control. (E–F) Immunoblot analysis of the isolated MA-10 subcellular fractions identifying mitochondrial matrix protein HSP60 (E) and peroxisomal matrix protein catalase (CAT) (F). GAPDH is used as a loading control. M, the protein marker, indicated in kDa; Total, whole cell lysate; Cyto, cytosolic fractions; Mito, mitochondrial-enriched fraction.

Immunofluorescent staining was performed to detect the endogenous distribution of SCPX and SCP2 in the MA-10 cell line, and DAPI dye was used to stain the nuclei. Endogenous SCPX was found to have a granular distribution in the cytosol, and was more likely to co-stain with anti-PMP70, a peroxisomal marker protein, compared to anti-VDAC1, a mitochondrial marker protein (Fig 5A). Immunoblotting was then performed on total protein extracts, cytosolic fractions and mitochondrial fractions from MA-10 cells using the antibodies specific for SCPX, COXIV (a mitochondrial control) and GAPDH (a whole cell lysate loading control). SCPX was present in the total protein extract and the cytosolic fraction, but it was not in the mitochondrial fraction (Fig 5B). However, endogenous SCP2 was found to have a granular distribution in co-staining with both anti-PMP70 and anti-VDAC1 proteins (Fig 5C). An immunoblot was performed using the antibodies for SCP2, VDAC1 (a mitochondrial control), and GAPDH (a whole cell lysate loading control). SCP2 was found in the total protein extract and in the mitochondrial fraction, but less in the cytosolic fraction (Fig 5D). These results suggest that SCPX is a peroxisomal protein, whereas SCP2 is likely to be strongly associated with the mitochondria. Furthermore, we validated the purity of the isolated subcellular fractions by immunoblot analyses using antibodies against the mitochondrial matrix protein HSP60 (Fig 5E) and the peroxisomal matrix marker protein catalase (CAT) (Fig 5F). The data obtained demonstrated that the mitochondrial matrix proteins remain dominantly in the mitochondrial, “mito”, subcellular fraction, and the peroxisomal matrix proteins are mostly found in the cytosolic, “cyto”, subcellular fraction.

### Peroxisomal localization of ECFP–SCPX/2 and cytoplasmic localization of SCPX/2–ECFP

**Fig 6:**
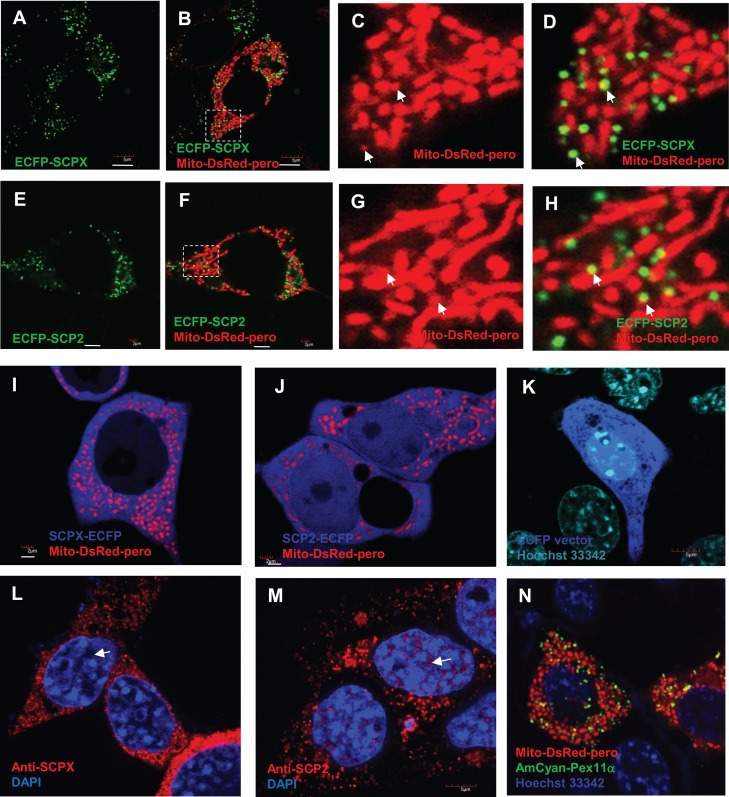
Live-cell imaging of the subcellular localization of SCPX, SCP2, and mito-DsRed-pero fluorescent fusion proteins in MA-10 cells via confocal laser microscopy. (A–H) Both ECFP–SCPX (A–D) and ECFP–SCP2 (E–H) fusion proteins were co-transfected with the mito-DsRed-pero. a dual peroxisomal and mitochondrial marker. The overlap indicates the localization of the ECFP–SCPX and ECFP-SCP2 proteins in the peroxisomes (shown by white arrows). Scale bar, 5 μm. (I–J) Both SCPX–ECFP (I) and SCP2–ECFP (J) proteins were co-transfected with the mito-DsRed-pero. When the peroxisomal-targeting signal was blocked, both SCPX–ECFP and SCP2-ECFP become widespread in the cytosol, and SCP2–ECFP is also distributed in the nucleus. Scale bar, 2 μm. (K) Transfection of the ECFP-N1 vector alone with nucleic acid staining using Hoechst 33342. Scale bar, 5 μm. (L–M) Immunofluorescent staining of the endogenous SCPX and SCP2 in MA-10 cells using SCPX- and SCP2-specific antibodies, where the nuclear staining of SCP2 is indicated by white arrows. (N) Colocalization of the mito-DsRed-pero fluorescent fusion protein with a peroxisomal membrane marker AmCyan-Pex11α, indicating the peroxisomal localization of mito-DsRed-pero, in addition to its mitochondrial localization.

Based on the preliminary findings that 22-NBD-cholesterol did not localize to the peroxisomes in the MA-10 cell line (Fig 1), and given that both SCPX and SCP2 are associated with peroxisomes *via* their PTS1 sequences whereas SCP2 is also closely related to mitochondria (Fig 5), further investigations were conducted to elucidate the subcellular localization of the SCPX and SCP2 proteins and, subsequently, the role of these proteins in the intracellular localization of 22-NBD-cholesterol. A fluorescent ECFP tag was fused to either the C-terminal or N-terminal end of both the SCPX and SCP2 proteins, and the subcellular localizations of these fusion proteins were then investigated via live-cell imaging using confocal laser microscopy. The C-terminal protein fusion creates the SCPX–ECFP and SCP2–ECFP fluorescent fusion proteins, blocking the peroxisomal-targeting PTS1 signal, while the N-terminal fusion creates the ECFP–SCPX and ECFP–SCP2 fusion proteins, blocking the predicted mitochondrial targeting sequence. MA-10 cells were co-transfected with mito-DsRed-pero and one of the fluorescent fusion proteins mentioned previously, and the intracellular localization of the fluorescently tagged proteins was visualized using a confocal laser microscope. It was observed that both SCPX (Fig 6A–6D) and SCP2 (Fig 6E–6H) were targeted to the peroxisomes, when the mitochondrial targeting sequence was obstructed. When the peroxisomal-targeting signal was blocked, it was found that the SCPX protein became widespread in the cytosol, but not in the nucleus (Fig 6I), while the SCP2 protein became a soluble protein found in both the cytosol and nucleus (Fig 6J). In addition, the fluorescent protein made from the empty vector ECFP-N1 alone was found to be distributed both in the cytosol and nucleus (Fig 6K), which seems to be inconclusive. However, immunofluorescent staining of the two endogenous proteins, SCPX and SCP2, indicated a nuclear localization for SCP2 (Fig 6L and 6M). The mito-DsRed-pero fluorescent protein was found to localize both at peroxisomes, as shown by its colocalization with the peroxisomal marker PEX11α, and mitochondria (Fig 6N). The molecular dissection of the signal sequences found in SCPX and SCP2 demonstrates that the peroxisomal-targeting PTS1 signal is sufficiently strong to ensure the peroxisomal localization of the proteins; however, the N-terminal mitochondrial targeting sequence is not powerful enough to localize the SCPX and SCP2 proteins to the mitochondria.

### Putative mitochondrial targeting sequence lacks potency to direct the fusion proteins to mitochondria

**Fig 7:**
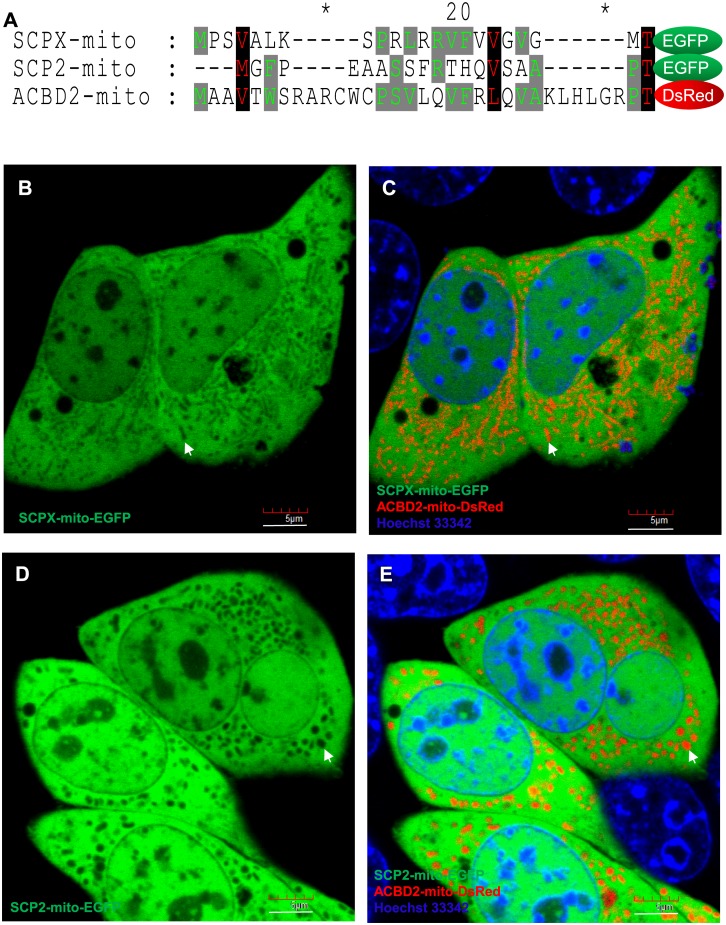
The presequences of SCPX and SCP2 show no role in mitochondrial targeting. (A) Sequence alignment of the predicted mitochondrial targeting sequences of SCPX (SCPX-mito) and SCP2 (SCP2-mito) compared to ACBD2 (ACBD2-mito), a known mitochondrial protein. (B-E) Live-cell imaging of the subcellular localization of SCPX-mito-EGFP (B-C) and SCP2-mito-EGFP (D-E) fluorescent fusion proteins (green) with ACBD2-mito-DsRed as a positive control (red). White arrow, indicating unstained mitochondria in B and D and the same mitochondria stained with ACBD2-mito-DsRed in C and E, respectively. Scale bar, 5 μm.

Several previous reports have shown or mentioned that the N-terminal sequence of pro-SCP2 contains a putative mitochondrial targeting sequence [14,17,36]. A sequence alignment of the predicted mitochondrial targeting sequences from SCPX (SCPX-mito), SCP2 (SCP2-mito) and ACBD2 protein (ACBD2-mito; used as the mitochondrial targeting sequence control), demonstrate the considerable similarities in the primary sequences of SCPX-mito and SCP2-mito compared to known mitochondrial proteins (Fig 7A). To further characterize the function of the putative mitochondrial targeting sequence and whether it is capable of targeting SCPX and SCP2 to the mitochondria, each mitochondrial targeting sequence was cloned to make the florescent fusion proteins SCPX-mito-EGFP and SCP2-mito-EGFP. The subcellular distributions of these fluorescent fusion proteins were then investigated using a confocal laser microscope with ACBD2-mito-DsRed, previously shown to be targeted solely to mitochondria, as a positive control. It was observed that neither SCPX-mito-EGFP (Fig 7B and 7C) nor SCP2-mito-EGFP (Fig 7D and 7E) was targeted to the mitochondria, marked by the ACBD2-mito-DsRed. It is likely true that the presequences play a role in assisting C-terminal PTS1-mediated peroxisomal targeting [36].

### Protein homology modeling and small molecular docking studies reveal the SCP2 domain in SCPX and SCP2 binds 22-NBD-cholesterol and cholesterol in a nonspecific manner

**Fig 8:**
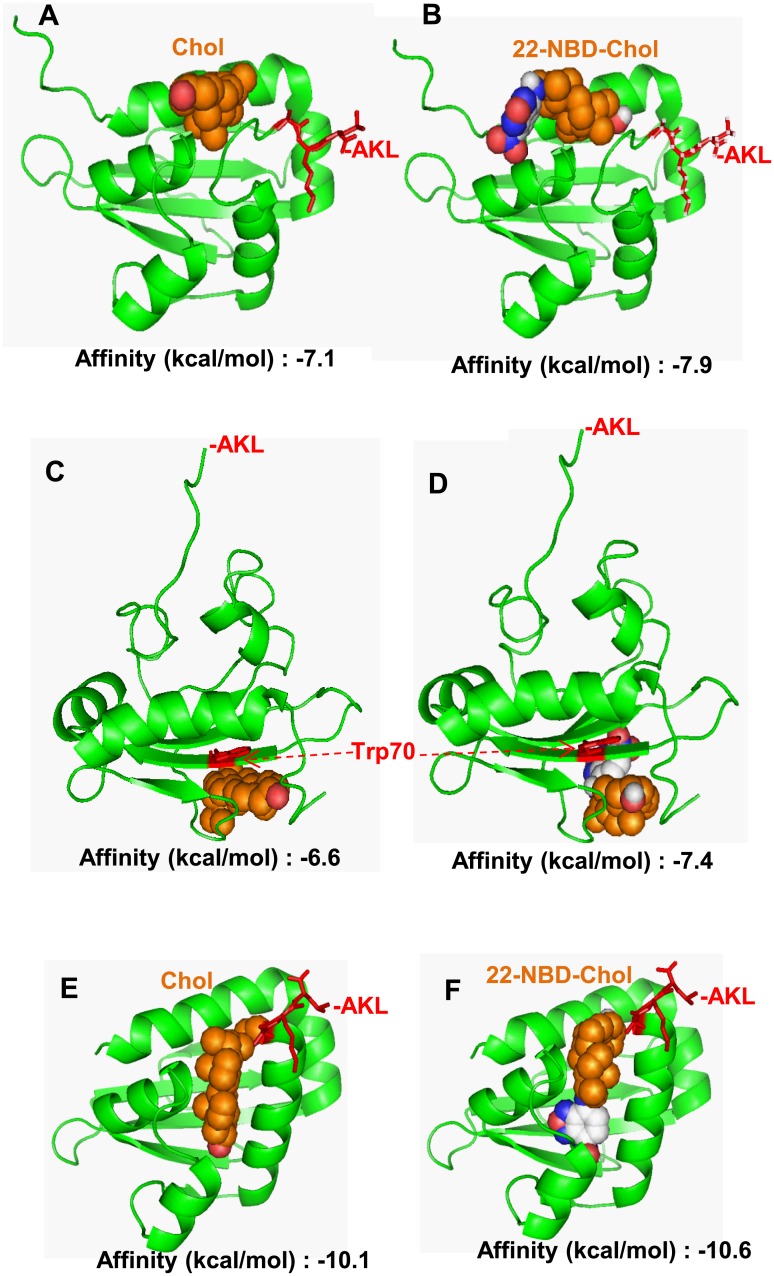
Homology modelling of the mouse SCP2 domain and molecular docking studies of SCP2 with its potential ligands, cholesterol (Chol) and 22-NBD-cholesterol (22-NBD-Chol). (A–B) Molecular docking study using homology models based on the X-ray crystallography structure of the rabbit SCP2 (PBD: 1C44). (C–D) Molecular docking study using homology models based on the NMR structure of the human SCP2 (PBD: 1QND). (E–F) Molecular docking study using homology models based on the X-ray crystallography structure of the ligand-bound SCP2-like domain of human peroxisomal multifunctional enzyme type 2 (MFE-2; PDB: 1IKT). Cholesterol (Chol), 22-NBD-cholesterol (22-NBD-Chol), the Trp70 residue responsible for binding cholesterol, and the peroxisomal-targeting PTS1 signal (-AKL) are indicated. The calculated docking affinity (kcal/mol) from Autodock-Vina is indicated under each corresponding conformation.

Putative 3D structures of the murine SCP2 domain from both the SCPX and SCP2 were predicted *via* Swiss-Model, an optimized automated comparative protein modeling server, using the coordinates of the X-ray crystallography structure of rabbit sterol carrier protein-2 (PDB accession number: 1C44) (Fig 8A and 8B) and the NMR structure of human sterol carrier protein-2 (PDB accession number: 1QND) (Fig 8C and 8D), both of which obtained from the Brookhaven Protein Database (36, 37). The Trp70 amino acid that is near the region of cholesterol binding is highlighted [7]. A homology model of the SCP2 domain was also obtained based on the SCP2 domain of human peroxisomal multifunctional enzyme type 2 (MFE 2) (PDB accession number: 1IKT) (Fig 8E and 8F). The molecular docking analyses using the homology models based on the rabbit and human SCP2 proteins (Fig 8A–8D) found that both cholesterol and 22-NBD-cholesterol bind outside of SCP2’s hydrophobic channel. Only the molecular docking study using the SCP2 domain of MFE-2 showed cholesterol and 22-NBD-cholesterol binding inside the channel (Fig 8E and 8F). These results suggest that the SCP2 domain binds cholesterol in a nonspecific manner, most likely on its surface, but it also has the potential to hold cholesterol within its hydrophobic channel with conformational changes under certain circumstances.

## Discussion

### 22-NBD-cholesterol and peroxisomal localization

22-NBD-cholesterol as the fluorescent cholesterol analog for fluorescence imaging of natural cholesterol may mimic the native distribution of free cholesterol, as previously shown in Leydig cells where the majority of peroxisomes were unstained for free cholesterol prior to the luteinizing hormone (LH) stimulation [37]. Although SCP2 favors a peroxisomal subcellular localization *via* its PTS sequence and it has a high binding affinity for 22-NBD-cholesterol [15,24], it does not necessarily cause the colocalization between 22-NBD-cholesterol and peroxisomes. Nevertheless, 22-NBD-cholesterol has been demonstrated in metabolic studies as a substrate of cholesterol-metabolizing oxidoreductase, the mammalian cytochrome P450 side-chain cleavage enzyme (CYP11A1), which is the key mitochondrial enzyme in the first step of steroid biosynthesis [38].

### Tissue-specific expression of SCPX and SCP2 in mouse testis and subcellular expression in MA-10 cells

The testis in male is capable of converting cholesterol into steroid hormones within the Leydig cells, which play an essential dominant role. Moreover, the SCP2 has been related to steroidogenesis *via* its affecting intracellular cholesterol trafficking [17]. Although SCP2 was believed to be a Leydig cell-specific protein [39], it was also found to be present in Sertoli cells [40]. Therefore, we performed additional exploratory experiments to investigate the presence and distribution of SCPX and SCP2 in the mouse testis. These findings would help determine the relevance of our high-resolution observations concerning the subcellular localizations of these proteins in MA-10 mouse Leydig tumor cells to animal models. In addition, Leydig cells are heavily involved in steroidogenesis, as they are the only cells in the male with the ability to synthesize and secrete testosterone [41]. Chronic luteinizing hormone (LH) treatment produces an increase in testosterone synthesis by Leydig cells in rats *in vivo*, which is accompanied by an increase in SCP2, although whether these observations are related remains to be determined [42]. In addition, acute LH stimulation leads to a rapid and transient increase in SCP2, and it has been proposed that SCP2 and peroxisomes play a role in testosterone biosynthesis before cholesterol is transported to the mitochondria [43]. Furthermore, LH deprivation leads to reductions in intraperoxisomal and total SCP2 in Leydig cells, as well as to a decrease in the synthesis and secretion of testosterone [21,43]. These findings all point towards Leydig cell peroxisomes and SCP2 playing a crucial role in testosterone production in the testes [21].

Both SCPX and SCP2 from our immunofluorescent staining are highly expressed in the interstitium of the testis, an area rich in Leydig cells, but both proteins have low levels of expression in the germ cells of the seminiferous tubules. This finding strengthens the hypothesis that SCPX and SCP2 are involved in steroidogenesis, since Leydig cells are known to be the steroid-producing cells. Even though we recognize that there are huge differences between normal murine Leydig cells and the MA-10 mouse Leydig tumor cell line, the cell line was selected for further study of the subcellular distribution of SCPX and SCP2 because it is a well-established model to study the cellular and molecular mechanisms underlying hormone-dependent Leydig cell steroidogenesis [27,29,44–46]. Although not much work has been done concerning SCP2 in the MA-10 cell line, a previous study has used the MA-10 cells to study SCP2’s role in steroidogenesis, except that study focused on isolated mitochondria instead of entire cells [47]. From our preliminary RNA-seq data performed on the whole transcriptome of the cells [27], we found that transcripts for both *Scpx* and *Scp2* demonstrate low expression in MA-10 cells. However, protein levels of these two proteins seem high enough to be detected during immunoblotting analysis and immunofluorescent staining, where it was found that the proteins are strongly associated with peroxisomes, as well as mitochondria. These findings align with previous speculations that testicular steroid synthesis in mitochondria is coupled with the peroxisomes, based on observations that LH treatment or LH deprivation causes an increase or decrease, respectively, in the number of peroxisomes in the cells [48].

### Dual or multiple intracellular localization of SCPX and SCP2

We originally hypothesized that both SCPX and SCP2 would be targeted to both the peroxisomes and mitochondria and bind cholesterol to assist in mitochondrial cholesterol transport during steroid biosynthesis. We theorized that the N-terminal fusion of the ECFP fluorescent tag to the SCPX and SCP2 proteins would lead to their localization to the peroxisomes, as the fluorescent tag would block the mitochondrial targeting sequence. Our results aligned with this hypothesis, as ECFP–SCPX and ECFP–SCP2 were found to co-localize with mito-DsRed-pero at the peroxisomes, demonstrating the targeting of ECFP–SCPX and ECFP–SCP2 to this organelle. These findings were in agreement with the presence of a peroxisomal targeting PTS1 signal on the C-terminal end of the proteins [25], and they show that the PTS1 is strong enough to target SCPX and SCP2 solely to the peroxisomes. Previous studies have found that although the majority of SCP2 is found in the peroxisomes, significant amounts are also detected in the mitochondria, endoplasmic reticulum, and cytosol [14–16]. However, we noted that ECFP–SCP2 appeared to localize exclusively to the peroxisomes when the N-terminal sequence was obstructed, suggesting that the N-terminal sequence of SCP2 might play a role in targeting the protein to these other organelles. Although SCPX also contains a putative mitochondrial targeting sequence at its N-terminus, previous studies have found it to be localized exclusively to the peroxisomes [17], aligning with our observation that ECFP–SCPX was only found in the peroxisomes.

We also hypothesized that the fusion of the ECFP tag to the C-terminal end of SCPX and SCP2 would lead to the localization of these proteins to the mitochondria, since the fluorescent tag would block the peroxisomal-targeting signal. This hypothesis was based on the observation that the N-termini of these proteins demonstrate characteristics typical of a mitochondria-targeting sequence [26,49]. Although SCPX has not been detected in the mitochondria, SCP2 has been identified in the mitochondria through studies using immunoblots of mitochondrial fractions [50], immunogold electron microscopy [16], and immunofluorescence microscopy [51]. However, our results deviated from expectations, since SCPX–ECFP and SCP2–ECFP became soluble proteins distributed throughout the cytoplasm, with SCP2–ECFP also being dispersed in the nucleus. Possible explanations for this observation could be that the mitochondrial targeting sequence may not be strong enough to target the SCPX and SCP2 proteins solely to the mitochondria, or that perhaps a different signalling sequence located elsewhere is responsible for the mitochondrial localization of the protein.

The widespread localization of the SCPX-mito-EGFP and SCP2-mito-EGFP fusion proteins in the cytosol and nucleus, but not in the mitochondria, confirmed that the mitochondrial targeting sequences of SCPX and SCP2 alone are not capable of targeting these proteins to the mitochondria. These results were compared to the positive control of ACBD2-mito-DsRed, a presequence known to target the ACBD2 protein to mitochondria. Therefore, it is evident that the mitochondrial targeting sequences of SCPX and SCP2 do not possess the potency needed to target the proteins to mitochondria.

### SCP2 binding of cholesterol and 22-NBD-cholesterol

SCP2 possesses a single cholesterol molecule binding site with a high affinity for cholesterol [24]. However, there have been no reports about the isolated native SCP2 in complex with cholesterol, indicating that either SCP2 does not bind cholesterol, or that the isolation process results in the loss of the bound cholesterol [14].

Our SCP2 homology modeling of the mouse SCP2 domain and molecular docking studies of cholesterol and 22-NBD-cholesterol suggest that SCP2 binds both cholesterol and 22-NBD-cholesterol on its surface with no dramatic difference in terms of binding affinity. In addition, these results suggest that SCP2 possesses the potential to embrace a ligand within its hydrophobic lipid-binding channel under certain circumstances, which may be consistent with the conformational plasticity of the protein [52]. This nonspecific binding of cholesterol or 22-NBD-cholesterol by SCP2 may represent that the cholesterol molecule being held in a position on the protein where it could easily be lost to other molecules during transport. In addition, SCP2 has one shared binding site for cholesterol, long-chain fatty acids and long-chain fatty acyl-CoAs, as well as a second specific binding site for long-chain fatty acids and long-chain fatty acyl-CoAs, but not cholesterol [24]. From two very recent reports, hepatic cholesterol accumulation was observed in mouse SCP2 knockout models [22,23]. It is clear that SCP2 plays a role in nonvesicular cholesterol transport between intracellular membranes in a nonspecific manner, and that it has a potential role in replenishing mitochondrial cholesterol pools depleted by steroid hormone synthesis in steroidogenic tissues.

## Conclusions

In conclusion, SCPX and SCP2 are present in MA-10 cells and in mouse testicular interstitial Leydig cells; SCPX and SCP2 are targeted to the peroxisomes by the C-terminal PTS1; the N-terminal mitochondrial targeting sequence does not target SCPX and SCP2 solely to the mitochondria; and cholesterol binds to the surface of SCP2 rather than in the hydrophobic lipid-binding pocket.

Overall, it appears that SCP2 present in the peroxisomes likely does not specifically bind cholesterol. However, the SCP2 outside the peroxisomes could bind cholesterol in a non-specific manner, which could explain the reason behind the initial observations that 22-NBD-cholesterol did not localize to the peroxisomes. In addition, these findings contribute to our understanding of intracellular cholesterol transport during steroid biosynthesis *via* SCP2, and it could also help uncover the roles of SCP2 in diseases involving lipid abnormalities.
